# Biya River Virus, a Novel Hantavirus of the Eurasian Water Shrew (*Neomys fodiens*) in Russia

**DOI:** 10.3390/v17111499

**Published:** 2025-11-12

**Authors:** Liudmila N. Yashina, Sergey A. Abramov, Ekaterina M. Luchnikova, Natalia A. Smetannikova, Tatiana V. Tregubchak, Tamara A. Dupal, Anton V. Krivopalov, Evgenia D. Vdovina, Richard Yanagihara

**Affiliations:** 1 State Research Center of Virology and Biotechnology Vector, 630559 Koltsovo, Russia; smetannikova@vector.nsc.ru (N.A.S.); tregubchak_tv@vector.nsc.ru (T.V.T.); 2 Institute of Systematics and Ecology of Animals SB RAS, 630091 Novosibirsk, Russia; terio@eco.nsc.ru (S.A.A.); dupalgf54@gmail.com (T.A.D.); krivopalov@gmail.com (A.V.K.); 3 Department of Ecology and Environmental Management, Institute of Biology, Ecology and Natural Resources, Kemerovo State University, 650099 Kemerovo, Russia; lut@yandex.ru (E.M.L.); vdovinae26@gmail.com (E.D.V.); 4 Department of Pediatrics, John A. Burns School of Medicine, University of Hawaii at Manoa, Honolulu, HI 96813, USA

**Keywords:** *Hantaviridae*, hantavirus, mobatvirus, shrew, evolution, Russia

## Abstract

*Hantaviridae* (order *Bunyavirales*) is a family of negative-sense, single-stranded RNA viruses. To date, several genetically distinct hantaviruses have been found in the same species of shrews and moles. In this report, we describe Biya River virus (BIRV), a novel hantavirus detected in the Eurasian water shrew (*Neomys fodiens*), the principal host of Boginia virus (BOGV). Genetic analysis of the complete L- and M-genomic segments and partial S-genomic segments showed that BIRV shared a common evolutionary origin with shrew-borne Altai (ALTV) and Lena (LENV) viruses, belonging to the *Mobatvirus* genus, and that BIRV was distantly related to BOGV and other shrew- and mole-borne orthohantaviruses. Ancient cross-species transmission of hantaviruses, with subsequent diversification within the Soricinae subfamily in Eurasia, might have shaped the evolutionary history of BIRV, ALTV, and LENV.

## 1. Introduction

*Hantaviridae* (order *Bunyavirales*), a family of negative-sense, single-stranded RNA viruses, has a genome comprising small (S), medium (M), and large (L) segments, which encode a nucleocapsid (N) protein and occasionally a nonstructural (NS) protein, envelope glycoproteins (Gn and Gc), and an RNA-dependent RNA polymerase (RdRp), respectively. More than 47 distinct species of hantaviruses identified in rodents, moles, shrews, and bats are classified into one of four genera (*Loanvirus*, *Mobatvirus*, *Orthohantavirus* and *Thottimvirus*) within the *Mammantavirinae* subfamily [1]. More than 50 additional presumptive hantaviruses remain unclassified.

Several rodent-associated hantaviruses (genus *Orthohantavirus*) are known human pathogens [2]. The initial hypothesis of strict co-evolution between rodent-borne hantaviruses and their hosts has been revised after the discovery of hantaviruses in shrews (family *Soricidae*), moles (family *Talpidae*), and bats (order Chiroptera) [3,4]. The evolutionary history of hantaviruses in moles, shrews, and bats is much more complex and includes cases of interspecies transitions and genome reassortment [5,6,7,8,9].

Studies of soricid- and talpid-borne hantaviruses have identified several pairs of significantly different hantaviruses that share a natural host. One such pair was discovered in the common shrew (*Sorex araneus*), the reservoir host of Seewis virus (SWSV), which is widespread across Western Europe to the Baikal region [10,11,12]. A new virus, named Altai virus (ALTV), was found co-circulating with SWSV in the same host species in Western Siberia and in European countries [6,13]. Also, the European mole (*Talpa europaea*), known as the natural host of Nova virus/*Mobatvirus novaense* (NVAV) [14], can harbor a second hantavirus, named Bruges virus/*Orthohantavirus brugesense* (BRGV), in European countries [15]. Moreover, the Iberian mole (*Talpa occidentalis*) has been shown to harbor NVAV, BRGV and Asturias virus (ASTV) in northwestern Spain [16]. These findings highlight the complexity of hantavirus–reservoir relationships. ALTV and LENV are members of the genus *Mobatvirus*, which includes most hantaviruses associated with bats. Based on these data, a hypothesis that they originate as a result of interspecies transfer has been proposed [6,17].

In Russia, studies of hantaviruses carried by shrews and moles, conducted mainly in Siberia and the Far East, have resulted in the identification of multiple hantaviruses, including Kenkeme virus/*Orthohantavirus kenkemeense* (KKMV), Artybash virus/*Orthohantavirus artybashense* (ARTV), ALTV, Lena virus/*Mobatvirus lenaense* (LENV), and Academ virus (ACDV) [6,15,17,18,19].

Boginia virus (BOGV) has been identified among Eurasian water shrews (*Neomys fodiens*) in Poland and Finland [5,20]. *N. fodiens* inhabits the forest zone from Western Europe to the Pacific coast. The presence of hantaviral antigens in the Eurasian water shrew was reported in the European part of Russia [21]. In this study we screened *N. fodiens* captured in Western Siberia for hantaviruses. We present the genetic and phylogenetic analyses of two highly distinct hantaviruses harbored by the same host, suggesting both co-evolution and a host species shift in their evolutionary history.

## 2. Materials and Methods

### 2.1. Trapping and Sample Collection

Shrews were trapped during August 2019–2020 and September 2022 in the Altai Republic and from June to August 2021–2025 in the Kemerovo Oblast of Western Siberia, Russia. All wildlife field operations, including the responsible treatment of animals, met the guideline requirements of the Order of the Russian High and Middle Education Ministry (No. 742 issued on 13 November 1984) and the Federal Law of the Russian Federation (No. 498-FZ issued on 19 December 2018). Field procedures and protocols were approved by the Institutional Animal Care and Use Committee of the Institute of Systematics and Ecology of Animals (Protocols No. 2020-02 issued on 14 May 2020, and No. 2021-01 issued on 28 April). The study did not involve endangered or protected species.

**Figure 1:**
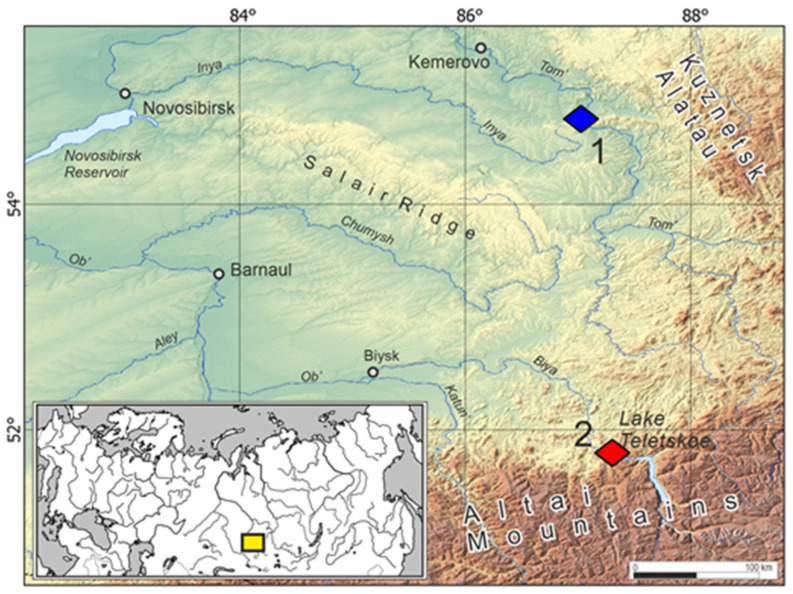
Map showing the locations of the collection sites in Western Siberia, where hantavirus-infected *Neomys fodiens* were captured. (1) Azhendarovo, (2) Teletskoye. Detected hantaviruses were Biya River virus (red) and Boginia virus (blue). The inset shows the location of the trapping area in Eurasia (yellow box). Map was generated using QGIS software (version 3.30.2; available at https://qgis.org). The base relief layer was obtained from the open-access service Maps-for-Free. Additional elements, including icons representing sampling sites, labels for geographic features, and an inset overview outline map, were added manually.

Collection sites (Figure 1) in the Altai Republic were located near Teletskoye Lake (51.79424 N/87.30447 E), and around the settlement Azhendarovo (54.76237 N/87.03094 E and 54.74537 N/87.02093 E) in the Kemerovo Oblast. Lung samples were collected aseptically and stored in RNAprotect^®^ (Qiagen, Hilden, Germany) before analysis.

### 2.2. RNA Extraction and RT-PCR Analysis

Total RNA was extracted from lung tissues, using the RNeasy MiniKit (Qiagen, Hilden, Germany), then reverse-transcribed, using the Expand reverse transcriptase (Roche, Basel, Switzerland) with universal oligonucleotide primer (OSM55, 5′–TAGTAGTAGACTCC–3′), designed from the conserved 3′ end of the S-, M-, and L-segments of hantaviruses. For initial screening by nested RT-PCR, previously described primers directed to a highly conserved region in the polymerase gene were used [22]. To confirm the taxonomic identity of hantavirus RNA-positive animals, genomic DNA was extracted from frozen lung tissue using the QIAamp DNA Mini Kit (Qiagen, Hilden, Germany), and the partial 426-nucleotide region of the cytochrome b gene of mitochondrial DNA (mtDNA) was amplified by PCR using universal primers: +14,115 (5′–CGAAGCTTGATATGAAAAACCATCGTTG–3′) and −14,532 (5′–CAGCCCCTCAGAATGATATTTGTCCAC–3′). Oligonucleotide primer sequences for PCR, specifically for BIRV, were designed from consensus regions of ALTV, LENV, and other hantaviruses. A partial S-segment was amplified using nested PCR with primers SAF20: 5′–AAACACATGGCRGATWTRARGCAAGG–3′ and S974R: 5′–TCNGGNGCHCHNGCAAANAHCCA–3′ and then SAF1: 5′–GGAGCAYAAAGGRAATAGGA–3′ and S974R. Short-sized amplicons were separated by electrophoresis on 1.2% agarose gels and purified using the QIAQuick Gel Extraction Kit (Qiagen, Hilden, Germany). DNA was sequenced directly using an ABI Prism 310 Genetic Analyzer (Applied Biosystems, Foster City, CA, USA).

Primers used for PCR amplification of the complete M-segment were M1F: 5′–TAGTAGTAGACTCCGCAARAA–3′ (the L-segment was amplified as two overlapping amplicons); L5-1: 5′–TTCTGCAGTAGTAGTAGACTCCG–3′; LR1: 5′–AACADTCWGTYC CRTCATC–3′; LF2: 5′–TGCWGATGCHACIAARTGGTC–3′; and LBR3: 5′–CTGCAGTAGTAGTAKRCTCCGG–3′. Long-sized amplicons were purified using the Mini Elute PCR Purification Kit (Qiagen, Hilden, Germany) and were used for library preparation with an NEBNext^®^ Ultra™ II FS DNA Library Prep with Sample Purification Beads (NEB, Hitchin, UK). Deep sequencing of libraries covering complete M and L segments of BIRV was performed on an Illumina MiSeq using the MiSeq reagent kit v3 (Illumina, San Diego, CA, USA). The full-length genomes were assembled de novo using MIRA assembly (version 4.9.6) and by the alignment of reads to known references with bwa-0.7.15 [23].

### 2.3. Genetic and Phylogenetic Analysis

Pairwise alignment and comparison of full-length coding regions of the M-, L-, partial S-, and L-segment nucleotide and amino acid sequences of hantaviruses from *N. fodiens*, captured in Russia, with representative shrew-, mole-, rodent-, and bat-borne hantaviruses, were performed, using MUSCLE in MEGA version 11 [24]. Phylogenetic trees were generated using the Maximum Likelihood approach with MEGA, under the best-fit general time-reversible model of nucleotide evolution with gamma-distributed rate heterogeneity and invariable sites (GTR + I + Г).

## 3. Results

### 3.1. Genetic Analysis

During 2019–2025, 64 Eurasian water shrews (*Neomys fodiens*) were captured at two localities in Western Siberia (Figure 1). *N. fodiens* were absent among shrews captured in locality Teletskoye in 2020 and 2022. RNAprotect^®^-preserved lung specimens were initially analyzed for hantavirus RNA by nested RT-PCR using oligonucleotide primers directed at the RdRp gene. Hantaviral RNA was detected in lung tissues from 4 of 64 Eurasian water shrews (Table 1). In all studied cases, cytochrome b typing matched morphological specification of hantavirus RNA-positive *N. fodiens*.

Genetic analysis of the partial L-segment sequences showed that two significantly different hantaviruses were detected in 4 of 64 *N. fodiens* tested (Table 1). One of these viruses, detected in three Eurasian water shrews captured at the locality Azhendarovo in Kemerovo Oblast, was BOGV, previously found in the same species in Poland and Finland [5,24]. The 346-nucleotide fragments of the L segment of BOGV showed 82.7–85.0% nucleotide (98.3% amino acid) similarity with BOGV strain 2074 from Poland. The genetic divergence between Siberian BOGV strains was 3.4–8.1% for nucleotides, while amino acid sequences were identical.

A separate genetically distinct hantavirus was identified in one of two *N. fodiens*, captured near the source of the Biya River, Teletskoye Lake locality, Altai Republic. This new hantavirus was designated Biya River virus (BIRV). The difference in the partial L-segment nucleotide and amino acid sequences from other representative hantaviruses exceeded 21.8% and 11.0% divergence between BIRV and the new BOGV strains from Siberia was 28.9–33.3% and 33.1%, respectively. The BIRV-positive Eurasian water shrew sample was subjected to full-genome sequencing. The full-length M and L segments of the BIRV genome were obtained. Despite multiple attempts, we were unable to obtain the full-length S segment; only a partial 485-nucleotide region of the N protein gene was obtained.

The partial S-segment sequence of BIRV (prototype strain Biya-Nf215/Russia/2019) displayed considerable divergence (>23.9% nucleotide and >35.0% amino acid) from other hantaviruses and was most closely related to strain HV/SC/C7-49.2/2022, recently discovered in the De Winton’s shrew (*Chodsigoa hypsibia*) in China [25].

The complete 3712-nucleotide M segment of BIRV contained a single ORF (positions 101–3523) encoding the 1140-amino acid glycoprotein precursor of the Gn and Gc glycoproteins, separated by a WAATA pentapeptide at position 649–653. The same motif was found in hantavirus HV/SC/C7-49.2/2022. Analysis of the complete M-coding sequence revealed more than 26.9% nucleotide and 22.6% amino acid sequence differences between BIRV and the most closely related HV/SC/C7-49.2/2022 and considerable divergence from other representative hantaviruses both at the nucleotide (>32.4%) and amino acid (>35.4%) levels. The M-segment sequence of BIRV showed 59.6% amino acid divergence from the partial 795-nucleotide M-segment sequence of BOGV (strain 2074), available in GenBank.

The complete 6537-nucleotide L segment of BIRV encoded an RdRp protein of 2146 amino acids. Pairwise alignment and comparison of the BIRV L segment with representative hantaviruses belonging to the four genera of the *Mammantavirinae* subfamily showed considerable divergence, ranging between 23.7 and 30.7% and 12.6 and 38.8% at the nucleotide and amino acid level, respectively.

The level of difference in the amino acid sequences of the partial N protein and GP encoded by the S and M segments from other hantavirus species was more than 35.0% and 22.6%, respectively, which meets the criteria for a new species and confirms the novelty of BIRV (Supplementary Table S1).

### 3.2. Phylogenetic Analysis

**Figure 2:**
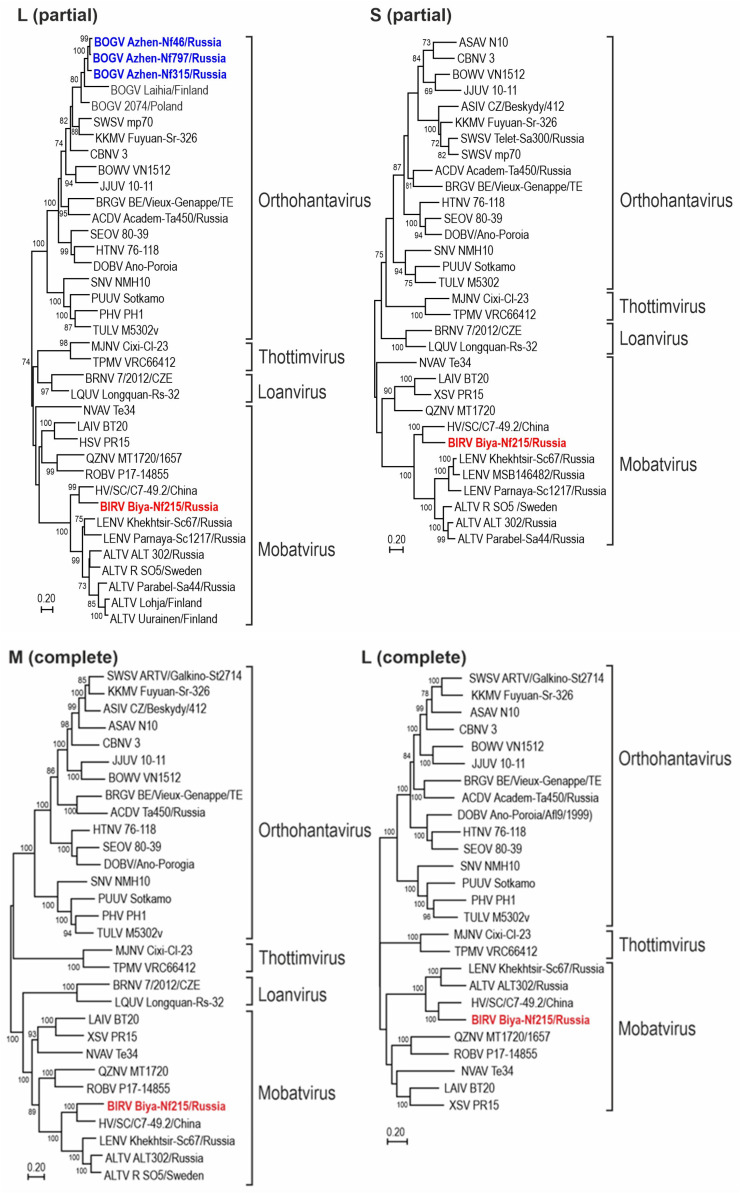
Phylogenetic trees, generated by maximum-likelihood methods, were based on the alignment of the 353-nucleotide (L partial) L-segment sequences, and 485-nucleotide (S partial) S-segment (**top**); the complete coding regions of the M (complete) and L (complete) segment sequences (**bottom**) of newfound BIRV and other representative hantaviruses. Bootstrap values (>70%) are shown at relevant nodes. The scale bar indicates the nucleotide substitutions per site. Colors (red and blue bold lettering) highlight newfound hantaviruses. Phylogenetic positions of BIRV are shown in relation to shrew- and mole-borne hantaviruses: ALTV (Altai virus), LENV (Lena virus/*Mobatvirus lenaense*), SWSV (Seewis virus), ASIV (Asikkala virus/*Orthohantavirus asikkalaense*, KKMV (Kenkeme virus/*Orthohantavirus kenkemeense*), BOGV (Boginia virus), HV/SC/C7-49.2 (*Hantaviridae* sp.), ASAV (Asama virus/*Orthohantavirus asamaense*), NVAV (Nova virus/*Mobatvirus novaense*), BRGV (Bruges virus/*Orthohantavirus brugesense*), ACDV (Academ virus), TPMV (Thottapalayam virus/*Thottimvirus thottapalayamense*), MJNV (Imjin virus/*Thottimvirus imjinense*), JJUV (Jeju virus/*Orthohantavirus jejuense*), CBNV (Cao Bằng virus/*Orthohantavirus caobangense*), and BOWV (Bowé virus/*Orthohantavirus boweense*). Also shown are representative rodent-borne hantaviruses, including SNV (Sin Nombre virus/*Orthohantavirus sinnombreense*), PHV (Prospect Hill virus/*Orthohantavirus prospectense*), TULV (Tula virus/*Orthohantavirus tulaense*), PUUV (Puumala virus/*Orthohantavirus puumalaense*), DOBV (Dobrava/Belgrade virus/*Orthohantavirus dobravaense*, HTNV (Hantaan virus/*Orthohantavirus hantanense*), and SEOV (Seoul virus/*Orthohantavirus seoulense*). Bat-borne hantaviruses include BRNV (Brno virus/*Loanvirus brunaense*), LAIV (Láibīn virus/*Mobatvirus laibinense*), XSV (Xuân Sơn virus/*Mobatvirus xuansonense*), QZNV (Quezon virus/*Mobatvirus quezonense*), LQUV (Lóngquán virus/*Loanvirus longquanense*), and ROBV (Robina virus/*Mobatvirus robinaense*). Additional information (virus strains, GenBank accession numbers, host species) is provided in Supplementary Table S2.

Phylogenetic trees, based on the coding regions of the full-length M and L segments and partial S segment of BIRV and the partial L segment of BOGV, were constructed using maximum-likelihood methods. In the tree, based on the partial L segment, the viral sequences recovered from Eurasian water shrews were placed into two genera (Figure 2). BOGV strains from Siberia and Europe occupied a separate branch within the genus *Orthohantavirus* and were grouped according to the geographical principle. The BIRV sequence was most closely related to strain HV/SC/C7-49.2/2022 from China and grouped with ALTV and LENV, previously identified in *Sorex* shrews sampled in northern Eurasia. The strain Biya-Nf215/Russia/2019 was most closely related to members of the *Mobatvirus* genus in the S-, M-, and L-segment phylogenetic trees (Figure 2).

## 4. Discussion

Here, we describe a new hantavirus, named BIRV, in the Eurasian water shrew, captured in Teletskoye Lake in the Altai Republic. BOGV sequences were detected in Eurasian water shrews captured 350 km away in the locality of Azhendarovo in Kemerovo Oblast. The capture sites in Western Siberia represent the foothill areas of Altai and Kuznetsk Alatau, respectively. Previously published data demonstrate a significant level of divergence in the genomes of hantaviruses detected in these foothill regions. Thus, a high level of partial L-segment divergence (up to 14.9% nucleotide) in each of the sites was established for the ACDV circulating among Siberian moles [26]. Similarly high divergence (up to 7.7% nucleotide) was shown for SWSV L sequences from Teletskoye Lake [11]. The exact trapping sites of BOGV in Azhendarovo were within 2 km, but partial L-segment sequences also demonstrated high divergences (up to 8.1%). Our data from L-segment phylogeny suggest that there are two sub-lineages of BOGV in *N. fodiens* from Azhendarovo. This finding supports our previous suggestion that foothill areas were colonized by several different routes of shrews from separate refugia [11].

The present-day distribution of mammals in northern Eurasia is largely the result of recolonization and dispersal of species in the period after the last glaciation [27]. In response to climate change, some mammal species have shifted their ranges in search of suitable conditions, persisting in large or small refugia [28,29]. The foothill regions of Altai and Kuznetsk Alatau are zones of secondary contacts of separately evolving lineages of moles and shrews and associated hantaviruses from different refugia [11,26].

The Eurasian water shrew, like many species of shrews, is widespread, but the animals prefer near-water biotopes. This explains the noticeable difference in the number of animals caught in the two localities. In the Teletskoye Lake area, a relatively high number of water shrews was observed along the shoreline, while in the meadow clearings among the forest, where the trapping site is located, the number of *N. fodiens* was comparatively low. In the community of eight species of shrew, *N. fodiens*, *S. araneus*, tundra shrew (*Sorex tundrensis*), taiga shrew (*Sorex isodon*), Laxman’s shrews (*Sorex caecutiens*), Eurasian pygmy shrew (*Sorex minutus*), Eurasian least shrew (*Sorex minutissimus*), and Siberian shrew (*Crocidura sibirica*), inhabiting this area, the predominant species is *S. araneus* (70.4%), while the proportion of the Eurasian water shrews is only 3.5% [30]. In 2019, 48 shrews including 2 *N. fodiens* (4.2%) were captured at this site, and these were absent among shrews captured in 2020 and 2022.

In Azhendarovo, the capture sites were located at the junction of the forest-steppe and taiga zones of the foothills of the Kuznetsk Alatau. The animals were caught in a floodplain meadow, an overgrown clearing in place of black taiga, and an ecotone area between the floodplain meadow and the overgrown clearing. In addition to the above-mentioned species from Lake Teletskoye, the flat-skulled shrew (*Sorex roboratus*) is added to the shrew community of Azhendarovo. The dominant species is also the common shrew (40.1%). The proportion of the Eurasian water shrew varies from 3.6% to 6.7% of the community [31]. The location of the sites and the longer period of capture determined the higher number of *N. fodiens* captured in this locality during 2021–2025.

The Eurasian water shrew, the host of BIRV and BOGV, is widespread in the forest zone of Eurasia from the British Isles to the Pacific Ocean [32]. The geographic range includes most of Europe, Siberian and far-eastern Russia, Sakhalin Island, North Korea, northwestern Mongolia, and China. BIRV was found in one locality of Western Siberia, while BOGV was detected in distant localities of its host geographic range, in Western Siberia and European countries, Poland, and Finland [5,20]. Based on the broad geographic range of other hantavirus pairs, ALTV and SWSV and LENV and ARTV, throughout the distribution of their hosts, we suggest that BIRV might also be widespread in the forest zone of Eurasia and co-circulate with BOGV among *N. fodiens*. Co-circulation of hantaviruses in the same host species also raises the distinct possibility of co-infection and reassortment or recombination as a mechanism for rapid evolutionary change.

Analysis of nearly the whole genome of BIRV demonstrated that it represents a new hantavirus species, which belongs to the *Mobatvirus* genus. Comparison of BIRV with available partial Siberian and European BOGV L- and M-segment sequences showed a significant difference between the two viruses. However, we found inconsistency during comparative sequence analysis of BIRV and BOGV (strain 2074) from Poland based on the available 783-nucleotide L-segment fragments. Two parts of this fragment demonstrated different levels of divergence with BIRV. The sequence corresponding to the 356- nucleotide fragments at positions 2980–3331 exhibited high nucleotide and amino acid sequence divergence (31.4% and 32.5%), while the adjacent 427-nucleotide fragment at positions 2541–2968 was much closer to BIRV (17.1% and 2.1%). Whether this means there might be evidence of recombination between BIRV and BOGV during co-infection warrants further investigation. Undoubtedly, analysis based on the partial L- and M-segment sequences is inadequate, and full-genomes of BOGV strains are needed to acquire a better understanding of the phylogenetic relationships and possible recombination events in the evolutionary history of BOGV.

Based on the complete M-, L- and partial S-segment phylogeny (Figure 2), BIRV and strain HV/SC/C7-49.2 from China shared a common ancestry with ALTV and LENV, together forming a sub-clade within the *Mobatvirus* genus. The natural host of HV/SC/C7-49.2, the De Winton’s shrew (*Chodsigoa hypsibius*), is endemic and widely distributed in central and southwestern China. The habitats of the natural hosts of ALTV (*S. araneus*), BIRV (*N. fodiens*), and LENV (*S. caecutiens*) overlap with each other and with *Chodsigoa hypsibius* in the Hengduan Mountains region, which ensures the possibility of contacts between species and interspecies transmission of associated viruses.

## 5. Conclusions

BIRV and BOGV represent genetically distinct hantaviruses that share the same host species. The finding of BIRV in *N. fodiens* supports the hypothesis that BIRV, ALTV, and LENV arose from ancient host-switching events from another reservoir host with subsequent diversification within the Soricinae subfamily in Eurasia.

## Figures and Tables

**Table 1:** Hantavirus RNA and hantavirus sequences in *Neomys fodiens* from Western Siberia, Russia.

		Positive/Tested		GenBank No.		
Capture Site	Year	Shrews	Virus Strain	S	M	L
Altai Republic, Teletskoye	2019	1/2	BIRV_Biya-Nf215	PQ355537	PQ355538	PQ355539
Kemerovo Oblast, Azhendarovo	2021	1/13	BOGV_Azhen-Nf315	-	-	PQ355534
	2022	2/17	BOGV_Azhen-Nf46	-	-	PQ355535
			BOGV_Azhen-Nf797	-	-	PQ355536
	2023	0/1	-	-	-	-
	2024	0/6	-	-	-	-
	2025	0/25	-	-	-	-

“-” sequences unavailable.

## Data Availability

GenBank accession numbers for newfound viruses are available in Table 1. The sequences will be released at the time of publication. Other presented data are available on request from the corresponding authors.

## Supplementary Materials

The following supporting information can be downloaded at: https://www.mdpi.com/article/10.3390/v17111499/s1, Table S1: Sequence distances (%) of the partial S (163 amino acids) and complete coding regions of the M (1140 amino acids) and L (2146 amino acids) segments of BIRV strain Biya-Nf215/Russia/2019 from Neomys fodiens and other rodent-, shrew-, mole- and bat-borne hantaviruses; Table S2: GenBank accession numbers, strains and host information for hantaviruses included in genetic and phylogenetic analysis.

## References

[1] Maes P., Adkins S., Alkhovsky S.V., Avšič-Županc T., Ballinger M.J., Bente D.A., Beer M., Bergeron E., Blair C.D., Briese T. (2019). Taxonomy of the order *Bunyavirales*: Second update 2018. Arch. Virol..

[2] Jonsson C.B., Figueiredo L.T., Vapalahti O. (2010). A global perspective on hantavirus ecology, epidemiology, and disease. Clin. Microbiol. Rev..

[3] Yanagihara R., Gu S.H., Arai S., Kang H.J., Song J.-W. (2014). Hantaviruses: Rediscovery and new beginnings. Virus Res..

[4] Zhang Y.Z. (2014). Discovery of hantaviruses in bats and insectivores and the evolution of the genus *Hantavirus*. Virus Res..

[5] Ling J., Sironen T., Voutilainen L., Hepojoki S., Niemimaa J., Isoviita V.M., Vaheri A., Henttonen H., Vapalahti O. (2014). Hantaviruses in Finnish soricomorphs: Evidence for two distinct hantaviruses carried by *Sorex araneus* suggesting ancient host-switch. Infect. Genet. Evol..

[6] Kang H.J., Gu S.H., Yashina L.N., Cook J.A., Yanagihara R. (2019). Highly divergent genetic variants of soricid-borne Altai virus (*Hantaviridae*) in Eurasia suggest ancient host-switching events. Viruses.

[7] Kang H.J., Bennett S.N., Dizney L., Sumibcay L., Arai S., Ruedas L.A., Song J.W., Yanagihara R. (2009). Host switch during evolution of a genetically distinct hantavirus in the American shrew mole (*Neurotrichus gibbsii*). Virology.

[8] Klempa B. (2018). Reassortment events in the evolution of hantaviruses. Virus Genes.

[9] Liphardt S.W., Kang H.J., Dizney L.J., Ruedas L.A., Cook J.A., Yanagihara R. (2019). Complex history of codiversification and host switching of a newfound soricid-borne orthohantavirus in North America. Viruses.

[10] Song J.-W., Gu S.H., Bennett S.N., Arai S., Puorger M., Hilbe M., Yanagihara R. (2007). Seewis virus, a genetically distinct hantavirus in the Eurasian common shrew (*Sorex araneus*). Virol. J..

[11] Yashina L., Abramov S., Gutorov V., Dupal T., Krivopalov A., Panov V., Danchinova G., Vinogradov V., Luchnikova E., Hay J. (2010). Seewis virus: Phylogeography of a shrew-borne hantavirus in Siberia, Russia. Vector-Borne Zoonotic Dis..

[12] Schlegel M., Radosa L., Rosenfeld U.M., Schmidt S., Triebenbacher C., Löhr P.W., Fuchs D., Heroldová M., Jánová E., Stanko M. (2012). Broad geographical distribution and high genetic diversity of shrew-borne Seewis hantavirus in Central Europe. Virus Genes.

[13] Yashina L.N., Abramov S.A., Zhigalin A.V., Smetannikova N.A., Dupal T.A., Krivopalov A.V., Kikuchi F., Senoo K., Arai S., Mizutani T. (2021). Geographic distribution and phylogeny of soricine shrew-borne Seewis virus and Altai virus in Russia. Viruses.

[14] Kang H.J., Bennett S.N., Sumibcay L., Arai S., Hope A.G., Mocz G., Song J.-W., Cook J.A., Yanagihara R. (2009). Evolutionary insights from a genetically divergent hantavirus harbored by the European common mole (*Talpa europaea*). PLoS ONE.

[15] Laenen L., Vergote V., Kafetzopoulou L.E., Wawina T.B., Vassou D., Cook J.A., Hugot J.P., Deboutte W., Kang H.J., Witkowski P.T. (2018). A novel hantavirus of the European mole, Bruges virus, is involved in frequent Nova virus coinfections. Genome Biol. Evol..

[16] Gu S.H., Miñarro M., Feliu C., Hugot J.-P., Forrester N.L., Weaver S.C., Yanagihara R. (2023). Multiple lineages of hantaviruses harbored by the Iberian mole (*Talpa occidentalis*) in Spain. Viruses.

[17] Yashina L.N., Kartashov M.Y., Wang W., Li K., Zdanovskaya N.I., Ivanov L.I., Zhang Y.Z. (2019). Co-circulation of distinct shrewborne hantaviruses in the far east of Russia. Virus Res..

[18] Kang H.J., Arai S., Hope A.G., Cook J.A., Yanagihara R. (2010). Novel hantavirus in the flat-skulled shrew (*Sorex roboratus*). Vector Borne Zoonotic Dis..

[19] Arai S., Kang H.J., Gu S.H., Ohdachi S.D., Cook J.A., Yashina L.N., Tanaka-Taya K., Abramov S.A., Morikawa S., Okabe N. (2016). Genetic diversity of Artybash virus in the Laxmann’s shrew (*Sorex caecutiens*). Vector Borne Zoonotic Dis..

[20] Gu S.H., Markowski J., Kang H.J., Hejduk J., Sikorska B., Liberski P.P., Yanagihara R. (2013). Boginia virus, a newfound hantavirus harbored by the Eurasian water shrew (*Neomys fodiens*) in Poland. Virol. J..

[21] Tkachenko E.A., Ivanov A.P., Donets M.A., Miasnikov Y.A., Ryltseva E.V., Gaponova L.K., Bashkirtsev V.N., Okulova N.M., Drozdov S.G., Slonova R.A. (1983). Potential reservoir and vectors of haemorrhagic fever with renal syndrome (HFRS) in the USSR. Ann. Soc. Belg. Med. Trop..

[22] Klempa B., Fichet-Calvet E., Lecompte E., Auste B., Aniskin V., Meisel H., Barrier P., Koivogue L., Meulen J., Krüger D.H. (2007). Novel hantavirus sequences in shrew, Guinea. Emerg. Infect. Dis..

[23] Li H. (2013). Aligning sequence reads, clone sequences and assembly contigs with BWA-MEM. arXiv.

[24] Tamura K., Stecher G., Kumar S. (2021). MEGA11: Molecular evolutionary genetics analysis version 11. Mol. Biol. Evol..

[25] Cui X., Fan K., Liang X., Gong W., Chen W., He B., Chen X., Wang H., Wang X., Zhang P. (2023). Virus diversity, wildlife-domestic animal circulation and potential zoonotic viruses of small mammals, pangolins and zoo animals. Nat. Commun..

[26] Yashina L.N., Panov V.V., Abramov S.A., Smetannikova N.A., Luchnikova E.M., Dupal T.A., Krivopalov A.V., Arai S., Yanagihara R. (2022). Academ virus, a novel hantavirus in the Siberian mole (*Talpa altaica*) from Russia. Viruses.

[27] Pavelková Řičánková V., Robovský J., Riegert J. (2014). Ecological structure of recent and last glacial mammalian faunas in northern Eurasia: The case of Altai-Sayan Refugium. PLoS ONE.

[28] Provan J., Bennett K.D. (2006). Phylogeographic insights into cryptic glacial refugia. Trends Ecol. Evol..

[29] Marková S., Horníková M., Lanier H.C., Henttonen H., Searle J.B., Weider L.J., Kotlík P. (2020). High genomic diversity in the bank vole at the northern apex of a range expansion: The role of multiple colonizations and end-glacial refugia. Mol. Ecol..

[30] Shchipanov N.A., Litvinov Y.N., Sheftel B.I. (2008). Rapid method for estimating local biodiversity of a community of small mammals. Contemp. Probl. Ecol..

[31] Ilyashenko V.B., Luchnikova E.M., Skalon N.S., Grebentschikov I.S., Kovalevsky A.V. (2019). Long-term dynamics of small-mammal communities in anthropogenically disturbed territories in the south-east of West Siberia. IOP Conf. Ser. Earth Environ. Sci..

[32] Bannikova A.A., Lebedev V.S., Pavlinov I.Y., Lissovsky A.A. (2012). Order Eulipotyphla. The Mammals of Russia: A Taxonomic and Geographic Reference.

