# Supplementary material for: Biya River Virus, a Novel Hantavirus of the Eurasian Water Shrew (Neomys fodiens) in Russia
Source: Viruses. 2025 Nov 12;17(11):1499. doi: 10.3390/v17111499 (PMC12656937; doi:10.3390/v17111499)
Supplement: Supplementary file 1 [file viruses-17-01499-s001.zip › Supplementary Table S1_.pdf]

Supplementary Table S1. Sequence distances (%) of the partial S (163 amino acids) and complete coding regions of the M (1140 amino acids) and L (2146 amino acids) segments of BIRV strain Biya-Nf215/Russia/2019 from *Neomys fodiens* and other rodent-, shrew-, mole- and bat-borne hantaviruses.

Hantavirus Genus	Virus and Strain	S	M	L
Mobatvirus	HV_HV/SC/C7-49.2	35.0%<br>453 aa	22.6%<br>1137 aa	12.6%<br>2147 aa
	ALTV_ALT_302	46.8%<br>448 aa	36.4%<br>1136 aa	21.1%<br>2147 aa
	LENV_Khekhtsir-Sc67	48.7%<br>448 aa	36.5%<br>1136 aa	21.0%<br>2146 aa
	LAIV_BT20	53.5%<br>427 aa	45.0%<br>1127 aa	33.9%<br>2145 aa
	QZNV_MT1720/1657	54.7%<br>429 aa	47.4%<br>1133 aa	34.9%<br>2147 aa
Loanvirus	BRNV_7/2012/CZE	60.8%<br>423 aa	54.9%<br>1136 aa	37.3%<br>2144 aa
	LQUV_Rs-32	55.3%<br>423 aa	54.1%<br>1136 aa	26.3%<br>108 aa[a]
Thottimvirus	TPMV_VRC66412	65.0%<br>435 aa	56.6%<br>1121 aa	39.4%<br>2150 aa
	MJNV_Cixi-Cl-23	68.1%<br>436 aa	58.5%<br>1120 aa	39.7%<br>2149 aa
Orthohantavirus	BOGV_2074	–	60.1%<br>265 aa[a]	16.4%<br>260 aa[a]
	SWSV_ARTV/Galkino-St2714	62.5%<br>429 aa	54.5%<br>1139 aa	37.6%<br>2151 aa
	ASIV_ACZ/Beskydy/412	58.0%<br>429 aa	54.0%<br>1139 aa	29.8%<br>486 aa[a]
	KKMV_Fuyuan-Sr-326	61.2%<br>429 aa	55.2%<br>1139 aa	37.9%<br>2151 aa
	CBNV_TC-3	61.9%<br>428 aa	54.1%<br>1139 aa	38.3%<br>2151aa
	ASAV_N10	66.7%<br>433 aa	54.1%<br>1140 aa	38.2%<br>2056 aa
	HTNV_76-118	61.5%<br>429 aa	55.9%<br>1135 aa	37.7%<br>2151
	PUUV_Sotkamo	65.4%<br>433 aa	54.6%<br>1148 aa	38.8%<br>2156 aa

For virus names and hosts, refer to Supplementary Table S2.
–, sequence unavailable
aa, amino acids.
