# Supplementary material for: Biya River Virus, a Novel Hantavirus of the Eurasian Water Shrew (Neomys fodiens) in Russia
Source: Viruses. 2025 Nov 12;17(11):1499. doi: 10.3390/v17111499 (PMC12656937; doi:10.3390/v17111499)
Supplement: Supplementary file 1 [file viruses-17-01499-s001.zip › Supplementary Table S2_.pdf]

Supplementary Table S2. GenBank accession numbers, strains and host information for hantaviruses included in genetic and phylogenetic analysis.

Virus Name (Abbreviation)	Strain Name	Host species	S	M	L
Altai virus (ALTV)	ALT302	*Sorex araneus*	MK340902	MK340903	MT648514
	R SO5	*Sorex araneus*	ON720815	ON720819	ON720823
	Uurainen	*Sorex araneus*	–	–	KJ136623
	Parabel-Sa44	*Sorex araneus*	MT560057	–	MN815789
Mobatvirus lenaense [a] (LENV)	Khekhtsir-Sc67	*Sorex caecutiens*	MH499470	MH499471	MH499472
	Parnaya-Sc1217	*Sorex caecutiens*	MW505551	–	MW505552
	MSB146482	*Sorex caecutiens*	KM361043	–	–
Seewis virus (SWSV)	mp70	*Sorex araneus*	EF636024	–	EF636026
	Telet-Sa300	*Sorex araneus*	MN815797	–	–
Orthohantavirus artybashense (ARTV)	Galkino-St2714	*Sorex tundrensis*	MG888402	MG913806	MH499473
Orthohantavirus asikkalaense (ASIV)	ACZ/Beskydy/412	*Sorex minutus*	NC_043070	NC_043069	NC_043068
Orthohantavirus kenkemeense (KKMV)	MSB148794	*Sorex roboratus*	GQ306148	–	–
	Fuyuan Sr326	*Sorex roboratus*	NC_034559	NC_034565	NC_034560
Boginia virus (BOGV)	2074	*Neomys fodiens*	–	JX990966	JX990965
	Laihia	*Neomys fodiens*	–	–	KJ136642
Hantaviridae sp.	HV/SC/C7-49.2	*Chodsigoa hypsibia*	OQ451885	OQ451886	OQ451887
Orthohantavirus asamaense (ASAV)	N10	*Urotrichus talpoides*	EU929072	EU929075	EU929078
Mobatvirus novaense (NVAV)	Te34	*Talpa europaea*	NC_034464	NC_034470	NC_034465
Orthohantavirus brugesense (BRGV)	BE/VieuxGenappe	*Talpa europaea*	MK340905	OL871119	NC_034402
Academ virus (ACDV)	Academ-Ta450	*Talpa altaica*	MK340905	OL871119	PX270319
Thottimvirus thottapalayamense (TPMV)	VRC66412	*Suncus murinus*	NC_010704	NC_010708	NC_034564
Thottimvirus imjinense (MJNV)	Cixi-Cl-23	*Crocidura lasiura*	NC_034558	NC_034557	NC_034564
Orthohantavirus jejuense (JJUV)	10-11	*Crocidura shantungensis*	NC_034398	NC_034404	NC_034399
Orthohantavirus caobangense (CBNV)	TC-3	*Anourosorex squamipes*	NC_034484	EF543526	NC_034485
Orthohantavirus boweense (BOWV)	VN1512	*Crocidura douceti*	NC_034405	KC631783	NC_034407
Orthohantavirus sinnombreense (SNV)	NMH10	*Peromyscus maniculatus*	NC_005216	NC_005215	NC_005217
Orthohantavirus prospectense (PHV)	PH-1	*Microtus pennsylvanicus*	M34011	X55129	EF646763
Orthohantavirus tulaense (TULV)	M5302v	*Microtus arvalis*	NC_005227	NC_005228	NC_005226
Orthohantavirus puumalaense (PUUV)	Sotkamo	*Myodes glareolus*	NC_005224	NC_005223	NC_005225
Orthohantavirus dobravaense (DOBV)	Ano-Poroia	*Apodemus flavicollis*	NC_005233	NC_005234	NC_005235

Orthohantavirus hantanense (HTNV)	76-118	*Apodemus agrarius*	NC_005218	NC_005219	NC_005222
Orthohantavirus seoulense (SEOV)	80-39	*Rattus norvegicus*	NC_005236	NC_005237	NC_005238
Mobatvirus laibinense (LAIV)	BT20	*Taphozous melanopogon*	KM102247	KM102248	KM102249
Mobatvirus xuansonense (XSV)	PR15	*Hipposideros pomona*	KC688335	NC_078261	NC_078262
Mobatvirus quezonense (QZNV)	MT1720/1657	*Rousettus amplexicaudatus*	NC_034400	NC_034393	NC_034401
Mobatvirus robinaense (ROBV)	P17-14855	*Pteropus alecto*	NC_055633	NC_055634	NC_055632
Loanvirus brunaense (BRNV)	7/2012/CZE	*Nyctalus noctula*	KX845678	KX845679	KX845680
Loanvirus longquanense (LQUV)	Rs-32	*Rhinolophus sinicus*	NC_043126	NC_043127	NC_043125

[a]Virus names according to latest ICTV classification.

–, data unavailable or not used.
